# Chronic kidney diseases among homeless and slum dwellers in Accra, Ghana

**DOI:** 10.11604/pamj.2021.38.340.27106

**Published:** 2021-04-08

**Authors:** Ahmed Tijani Bawah, Foster Edufia, Fatima Nasara Yussif, Anastasia Adu, Yakubu Ayalsuma Yakubu

**Affiliations:** 1 Department of Medical Laboratory Sciences, School of Allied Health Sciences, University of Health and Allied Health Sciences, Ho, Ghana,; 2 Graduate Centre of Management, Cape Peninsula University of Technology, Cape Town, South Africa

**Keywords:** Homeless, slum dwellers, chronic kidney disease, head porters

## Abstract

**Introduction:**

chronic kidney disease is an important risk factor for cardiovascular-related morbidity and death. In Ghana, relatively little is known about the prevalence of chronic kidney disease (CKD) in homeless and slum dwellers in the major cities of the country. This study aimed at determining the prevalence of CKD among homeless people in Nima and Agbogbloshie, Accra, Ghana, and to evaluate the association between socio demographic characteristics and CKD.

**Methods:**

we recorded information on individuals' socio-demographic characteristics and anthropometric indices, and took blood samples from a total of 512 homeless participants for serum creatinine measurement. Renal function was estimated according to the 4-variable Modification of Diet in Renal Disease (MDRD) and Cockcroft–Gault (C-G) equations.

**Results:**

participants with normal serum creatinine (SCr), made up of 232 males and 280 females totaling 512 took part in the study. Those with normal glomerular filtration rate (GFR) were 86% and 84.6% by means of the C-G and MDRD equations respectively. According to the C-G formula, kidney damage and mild to severe renal insufficiency was found in 13.2% of the participants and 4 participants (0.8%) had renal failure. On the other hand, 15.4% of the participants were found to have some kidney damage and mild to severe renal insufficiency according to the MDRD formula with no participant suffering from kidney failure.

**Conclusion:**

the prevalence of CKD among the homeless Ghanaians was significant, especially among those with hypertension, diabetes and human immunodeficiency syndrome virus (HIV) infection.

## Introduction

Chronic kidney disease is the presence of reduced function of the kidneys lasting more than 3 months and characterized by estimated glomerular filtration rate (eGFR) of less than 60mL/minute per 1.73m^2^. It is also associated with presence of abnormalities in renal imaging, renal biopsy or urine sediments results [1]. Impaired renal function is linked to increased risk of anemia, disorders of bone mineralization, cardiovascular diseases and renal failure [2]. Homeless people with CKD face unique barriers to obtaining effective health care and so may seek help in emergency departments and other expensive alternatives [3,4]. Most homeless Ghanaians including female head porters (Kayayei) face difficulties in getting health care services in Accra due to lack of finances [5]. Besides, ineffective communication between health care professionals and patients as a result of language barriers limit most of these homeless migrants from seeking healthcare services from accredited health facilities. Consequently, some of these homeless migrants patronize the services of informal health care providers though a few of them may be holders of valid National Health Insurance Scheme (NHIS) card [5]. The prevalence of CKD is estimated to be between 10 and 13% globally [6,7] and 13.9% in sub-Saharan Africa [8]. In Ghana, CKD has been estimated to be 13.3% [9], however, very little is known about its prevalence among homeless people in Ghana, due to lack of surveillance systems for monitoring the health needs of the poor and homeless and hence insufficient data on risk factors for end stage renal disease (ESRD) [10]. We therefore carried out a survey of the prevalence of CKD among homeless and slum dwellers in two suburbs (Agbogbloshie and Nima) of Accra, Ghana, so as to provide base line information on the burden of this disease in this vulnerable group of our society.

## Methods

Selection of participants was done according to well-defined criteria as outline in our earlier publication on the prevalence of diabetes in homeless and slum dwellers in Accra, Ghana [11]. A total of 512 homeless subjects took part in the study comprising 232 males and 280 females with mean age of 38.3 ± 12.0 and 40.0 ± 10.4 respectively. Information on participants´ socio-demographic characteristics were recorded. Height was measured of participants not wearing shoes using a stadiometer to the nearest 0.5cm with the study participants standing upright and heels put together and the head in the horizontal plane. Weight was measured in kilograms using the Bioimpedance analyzer (BIA) (BSD01, Pure Pleasure, a division of the Stingray Group, Cape Town, South Africa). Body mass index (BMI) was calculated using the following formula: BMI = body weight (kg)/[height (m)]^2^ [11]. Five milliliters of venous blood and 10 ml of urine samples were taken from all participants into serum separator tubes and urine containers respectively. The blood samples were then centrifuged to obtain sera and both urine and serum samples were stored in several aliquots at -80°C until sample analysis. Serum and urine creatinine were determined using the Vitros dry chemistry analyzer (OrthoClinical Diagnostics, Johnson & Johnson, High Wycombe, UK). Urine albumin was determined using the dip-stick qualitative/semi-quantitative method (Urit Medical Electronic Co., Ltd, Guangxi, People´s Republic of China) following manufacturer´s instructions. Renal function was estimated using the Modification of Diet in Renal Disease (MDRD) equation [12] and the Cockcroft-Gault (C-G) equation, normalized for the body surface area (BSA) [13] while the classification of the stages of chronic kidney disease was based on markers of renal pathology (eGFR and the presence of albuminuria) [1,14]. Normal GFR was regarded as stage 1, mildly decreased was stage 2 while moderately decreased, severely decreased and kidney failure were regarded as stage 3, stage 4 and stage 5 respectively [15].

**Statistical analysis:** the data was first entered into Microsoft Office Excel 2007. We calculated prevalence estimates with the SAS 9.3 program (SAS Institute Inc., Cary, North Carolina). Proportions of those with renal insufficiency and stages of CKD were also calculated. Frequencies were reported as unweighted counts. The level of statistical significance was set at P < 0.05 for all tests and at 95% confidence interval.

**Ethics approval and consent to participate:** the study protocol was reviewed and approved by the institutional Research Ethics Committee of the University of Health and Allied Sciences, Ho, Ghana, protocol number: UHAS-REC A.4 [175]. All participants provided written informed consent and the procedure adopted conformed to the provisions of the Declaration of Helsinki (as revised in Fortaleza, Brazil, October 2013). Moreover, confidentiality was assured for all the information provided and personal identifiers were not included on questionnaire.

## Results

**Sociodemographic background of respondents:** the demographic characteristics of the study population representing homeless people in Accra is shown in Table 1. The participants in this study comprised 453 (88.5%) homeless Ghanaians and 59 (11.5%) non-Ghanaian homeless people. Among the Ghanaians, 373 (72.9%) originated from the northern regions of Ghana and the remaining 80 (15.6%) originated from the other regions of Ghana. Majority of the participants lived in the streets, kiosks, Shacks or other temporary structures in slums around Nima and Agbogbloshie in Accra for periods ranging from < 1 year (28.3%) to ≥ 5 years (7.0%). Most of the participants who hailed from the Northern parts of the country were head porters popularly referred to as Kayayei (plural), Kayayo (singular). Alcohol consumption and cigarette smoking among participants were very low; 3.1% and 0.8% respectively. A total of 31 (6.1%), 7.8% and 4.7% had medical history of diabetes hypertension and HIV respectively. None of the participants had tertiary education and 33.8% had no any formal education (Table 2) [11].

**Table 1:** demographic and clinical characteristics of study subjects (n=512)

Gender	Male n (%)	Female n (%)	Total (%)
	232 (45.3)	280 (54.7)	512 (100)
**Nationality**			
Ghanaians	205	248	453 (88.5)
Non-Ghanaians	27	32	59 (11.5)
**Ethnic origin**			
Northerners	169	205	373(72.9)
Southerners	37	43	80 (15.6)
Others	26	32	59 (11.5)
**Age (years)**			
20-29	59	106	165 (32.2)
30-39	79	114	193 (37.7)
40-49	55	43	98 (19.1)
50-59	24	12	36 (7.0)
60-69	12	5	17 (3.3)
≥70	3	0	3 (0.6)
**BMI (kg/m^2^)**			
<18.5	4	12	16 (3.1)
18.5-24.9	118	75	193 (37.7)
25-29.9	59	110	169 (33.0)
≥30	51	83	134 (26.2)
**Duration of homelessness (Years)**			
≤ 1	35	110	145 (28.3)
2	90	114	204 (39.8)
3	59	39	98 (19.2)
4	20	9	29 (5.7)
≥5	28	8	36 (7.0)
**Alcohol consumption**			
Yes	12	4	16 (3.1)
No	220	276	496 (96.9)
**Smoking**			
Yes	4	0	4 (0.8)
No	228	280	508 (99.2)
**Medical history**			
Diabetes	12	19	31 (6.1)
Hypertension	16	24	40 (7.8)
HIV	4	20	24 (4.7)
Normal	200	217	417 (81.4)
**Education level**			
No education	67	106	173 (33.8)
Primary	63	67	130 (25.4)
Middle/JHS	59	59	118 (23.0)
Voc/Tech/SHS/O'/A' Level	35	47	82 (16.0)
Tertiary	8	1	9 (1.8)

BP blood pressure, CVD cardiovascular disease, eGFR estimated glomerular filtration rate

**Table 2:** assessment of renal function of participants using different criteria

CKD classification	GFR (ml/min/1.73 m^2^)	Cockcroft-Gault n (%)	MDRD n(%)
Normal	≥ 60*	440 (86)	433 (84.6)
Stage 1	≥ 90✝	36 (7)	39 (7.6)
Stage 2	60-89.9✝	16 (3.1)	20 (3.9)
Stage 3	30-59.9	12 (2.3)	16 (3.1)
Stage 4	15-29	4 (0.8)	4 (0.8)
Stage 5	< 15	4 (0.8)	-

* With no kidney damage, ✝with kidney damage (Defined as presence of albuminuria, a urine albumin: creatinine ratio of > 2.0 mg/mmol for men or > 2.8 mg/mmol for women.

**Estimation of CKD among the participants:** estimation of renal function of the participants is presented in Table 2. According to the C-G formula, 14% of the homeless and slum dwellers were having renal impairment while 15.4% had renal impairment according to the MDRD formula. Classification of CKD revealed that 7% had stage 1, 3.1% had stage 2 while 2.3%, 0.8%, and 0.8% had stage 3, stage 4 and stage 5 respectively using the C-G formula. On the other hand, 7.6% had stage 1, while 3.9%, 3.1%, and 0.8% had stage 2, stage 3 and stage 4 respectively using the MDRD equation with none presenting with kidney failure (stage 5) (Table 2). About 7% and 7.6% of the participants were classified as stage 1 CKD [(eGFR ≥ 90 mL/min per 1.73 m^2^) and screened positive for albuminuria] according to C-G and MDRD equations respectively. For stage 2; [mildly reduced eGFR (60-89 mL/min per 1.73 m^2^) and presence of albuminuria], we recorded 3.1% and 3.9% using the C-G and MDRD equations respectively (Table 2). Characteristics of participants with various degrees of renal insufficiency is presented in Table 3. In all, 9.5% of males had stage 1 or 2 CKD as against 13.2% of females. Men with stage 3-5 CKD were 3.0% as compared to 4.0% of women. CKD was also stratified according to those below 40 years and those above 40 years. For those below 40 years, 9.8% had stage 1 or 2 renal impairment while 1.4% had stage 3-5 renal CKD. This is contrary to those above 40 years who had more subjects (10.4%) with stage 3-5 CKD but less subjects (7.8%) with stage 1-2 renal CKD. The result showed that those above 40 years were more likely to develop stage 3-5 CKD (p < 0.05). A total of 71 participants had diabetes, hypertension or HIV out of which 5.6% had stage 1 or 2 and 11.3% had stage 3-5 CKD showing that diabetes, hypertension and HIV predisposes a person to CKD (p < 0.05) (Table 3). There was no so much difference between C-G and MDRD in terms of classification of the CKD.

**Table 3:** prevalence of chronic kidney disease among homeless people in Accra

Group; stage of chronic kidney disease	No. of participants (512)	Prevalence % (95% CI)
**All Cockcroft-Gault (n=512)**		
Normal	440	86 (84.6-88.1)
Stage 1 or 2	52	10.1 (7.9-11.3)
Stages 3-5	20	3.9 (2.5-4.1)
**All (MDRD equation) (n = 512)**		
Normal	433	84.6 (84.7-88.3)
Stage 1 or 2	58	11.3 (8.8-13.6)
Stages 3-5	21	4.1 (3.1-5.1)
**Men (n = 232)**		
Normal	203	87.5 (85.1-89.7)
Stage 1 or 2	22	9.5 (7.8-11.9)
Stages 3 -5	7	3.0 (2.1-3.8)
**Women (n =280)**		
Normal	232	82.8 (85.3-89.1)
Stage 1 or 2	37	13.2 (7.5-11.5)
Stages 3-5	11	4.0 (2.3-4.9)
**Age 20 - 39 yr (n = 358)**		
Normal	318	88.8 (87.8-92.2)
Stage 1 or 2	35	9.8 (7.6-11.9)
Stages 3-5	5	1.4
**Age≥ 40 yr (n = 154)^a^**		
Normal	126	81.8 (87.2-92.3)
Stage 1 or 2	12	7.8 (6.6-11.2)
Stages 3-5	16	10.4 (0.7-2.7)
**Without diabetes, hypertension or HIV (n = 441)**		
Normal	398	90.2 (88.0-92.2)
Stage 1 or 2	35	8.0 (6.9-10.2)
Stages 3-5	8	1.8 (0.7-2.4)
**With diabetes, hypertension or HIV (n = 71)^a^**		
Normal	59	83.1 (80.7-85.4)
Stage 1 or 2	4	5.6 (NA)
Stages 3-5	8	11.3 (NA)

a Differences or associations significant at p < 0.05 for CKD classification of stages 3-5 CKD (moderately decreased to severely decreased and renal failure). NA; Not applicable

## Discussion

This study was aimed at determining the prevalence of CKD among homeless Ghanaians. Chronic kidney disease is a notable risk factor for death and cardiovascular-related morbidity [16]. Much attention has not been given to CKD in most sub-Saharan African countries including Ghana [17] though it is associated with increasing morbidity and mortality [18]. Homeless people with CKD often suffer from increased morbidity and mortality [3] and may not report to hospital early for treatment. The phenomenon of homelessness has assumed socio medical issue with high morbidity and mortality among them even in the developed world [19]. Most of the homeless people migrated from the 5 poorest regions of northern Ghana with few coming from the rest of the country and other neighboring countries. These homeless people usually live in dilapidated structures with no access to basic services with about 92% of those in Agbogloshie and 60% of those in Nima having no access to portable drinking water [19], and are prone to various illnesses which could eventually lead to their death [20]. Our study showed that 14% and 15.4% of homeless migrants living in slums around Nima and Agbogloshie in Accra, Ghana, are living with CKD using C-G and MDRD equations respectively. This is slightly higher than what had been reported in a previous study among the general population in Ghana [9]. Homeless people are more likely to engage in substance abuse and may also suffer from depression [3] which could have devastating effect on renal function. Another reason for the high prevalence of CKD in the homeless Ghanaians compared to what was reported previously in the general population is lack or non-renewal of their national health insurance scheme (NHIS) thus, the country is still struggling to achieve NHIS´s goal of universal health care [21]. The lack of valid NHIS cards makes it difficult for them to report to the hospital regularly and therefore are likely to develop renal disease than the general population. Scrap dealers in Agbogbloshie have been breaking down electronic wastes which emit toxic chemicals into the environment. Consequently, poisonous substances permeate the surrounding soil, water and air, posing serious health risks and could have effect on kidney function of inhabitants of the area and these explain why the prevalence of CKD in these homeless people is higher than the previously reported prevalence of (13.3%) among ordinary Ghanaians [9]. This is in consonance with a study in China which concluded that electronic waste dismantling activity has a negative impact on kidney function of those people with occupational exposure [22].

We report more women with moderate to severe kidney disease than men in line with previous report which stated that the proportion of women with pre dialysis CKD is higher than that of men [23]. Some authors have attributed this to overdiagnosis associated with the use of the eGFR equations and longer life expectancy of women [23]. Participants older than 40 years had higher prevalence of stages 3-5 CKD as compared to those below 40 years. This is in agreement with a previous study which found that CKD was higher in older people [24] and also in consonance with a previous study which reported that 17% of people above 60 years had an eGFR less than 60mL/min per 1.73m^2^ [25]. We identified CKD in 16.9% of the participants with hypertension and diabetes or HIV as against 9.8% of those without diabetes and hypertension or HIV. Though this is lower than what had been reported in South-Western Ghana [23], the fact that our results indicate higher prevalence among those with diabetes and hypertension re-emphasize the believe that diabetes and hypertension are important risk factors of renal impairment. However, the difference in our results and that of the study in South-Western Ghana could be due the differences in sample size. Another reason for the differences in the results could be due the fact that the incidence of diabetes among homeless Ghanaians was slightly lower than the general population [11] and since diabetes is a risk factor for CKD, renal diseases which are attributable to diabetes and hypertension may be lower in the homeless and slum dwellers. The limitation in this study is that family history of diabetes mellitus, hypertension and cardiovascular diseases could not be obtained, because participants were either not willing to or genuinely did not have such information.

## Conclusion

This study demonstrates substantial prevalence of renal impairment among homeless and slum dwellers especially among those with diabetes, hypertension and HIV. The findings have important inferences. Policy makers and stakeholders need to deliberate on programs and policies to address the need for social protection and support for this vulnerable group so as to curb the rising incidence of renal disease among the homeless in our society.

### What is known about this topic

- CKD is an important risk factor for cardiovascular-related morbidity and death;
- CKD is higher among people living with diabetes mellitus.

### What this study adds

- Electronic waste has effect on renal function;
- CKD is substantially high among homeless and slum dwellers.
